# High Source Levels and Small Active Space of High-Pitched Song in Bowhead Whales (*Balaena mysticetus)*

**DOI:** 10.1371/journal.pone.0052072

**Published:** 2012-12-26

**Authors:** Outi M. Tervo, Mads F. Christoffersen, Malene Simon, Lee A. Miller, Frants H. Jensen, Susan E. Parks, Peter T. Madsen

**Affiliations:** 1 Arctic Station, University of Copenhagen, Qeqertarsuaq, Greenland; 2 Greenland Climate Research Centre, Greenland Institute of Natural Resources, Nuuk, Greenland; 3 Institute of Biology, University of Southern Denmark, Odense M, Denmark; 4 Biology Department, Woods Hole Oceanographic Institution, Woods Hole, Massachusetts, United States of America; 5 Department of Biology, Syracuse University, Syracuse, New York, United States of America; 6 Zoophysiology, Department of Bioscience, Aarhus University, Aarhus C, Denmark; University of Lausanne, Switzerland

## Abstract

The low-frequency, powerful vocalizations of blue and fin whales may potentially be detected by conspecifics across entire ocean basins. In contrast, humpback and bowhead whales produce equally powerful, but more complex broadband vocalizations composed of higher frequencies that suffer from higher attenuation. Here we evaluate the active space of high frequency song notes of bowhead whales (*Balaena mysticetus*) in Western Greenland using measurements of song source levels and ambient noise. Four independent, GPS-synchronized hydrophones were deployed through holes in the ice to localize vocalizing bowhead whales, estimate source levels and measure ambient noise. The song had a mean apparent source level of 185±2 dB rms re 1 µPa @ 1 m and a high mean centroid frequency of 444±48 Hz. Using measured ambient noise levels in the area and Arctic sound spreading models, the estimated active space of these song notes is between 40 and 130 km, an order of magnitude smaller than the estimated active space of low frequency blue and fin whale songs produced at similar source levels and for similar noise conditions. We propose that bowhead whales spatially compensate for their smaller communication range through mating aggregations that co-evolved with broadband song to form a complex and dynamic acoustically mediated sexual display.

## Introduction

Whales rely on sound as the primary modality for communication, orientation and finding food [1]. Sound moves through water with high speed and, for lower frequencies, with little attenuation, which favours long-range social signaling [2]. The acoustic properties of a communication signal such as source level, directionality, frequency, bandwidth and duration, will greatly influence the type of information that can be communicated. Environmental sound propagation properties and ambient noise levels in addition to source parameters will define the range over which acoustic information can be relayed [3]. The active space of an acoustic signal is defined as the maximum range from the vocalizing animal where the sound level allows a conspecific to detect and decode the signal [4]–[6]. The active space has important implications for the evolution and function of acoustically mediated behaviour. To estimate the active space of a particular communication signal it is necessary to know the source level (defined as the sound level 1 m from the vocalising animal on the acoustic axis [7]), the frequency bandwidth, the sound attenuation of the signal through the habitat, the ambient noise and the hearing capabilities of the listener [5], [6].

Some animals have very small active spaces such as whispering moths that can only hear each other over a few centimetres [8]. Baleen whales, on the other hand, produce powerful signals at low frequencies [9], providing the basis for long range communication [10]. Blue whales (*Balaenoptera musculus*) and fin whales (*B. physalus*) produce simple narrowband songs [9] with the lowest frequencies and highest energy contents of any animal. Their songs and calls have dominant frequencies that range from 15 to 29 Hz[11]–[14] with mean source levels around 186 to 189 dB re 1 µPa (root-mean-square, rms) @ 1 m [12]–. The combination of high source levels and low sound frequencies, where little sound energy is lost due to absorption, results in active spaces of hundreds to thousands of km for blue and fin whales under natural ambient noise conditions [10], [14], [15].

**Figure 1:**
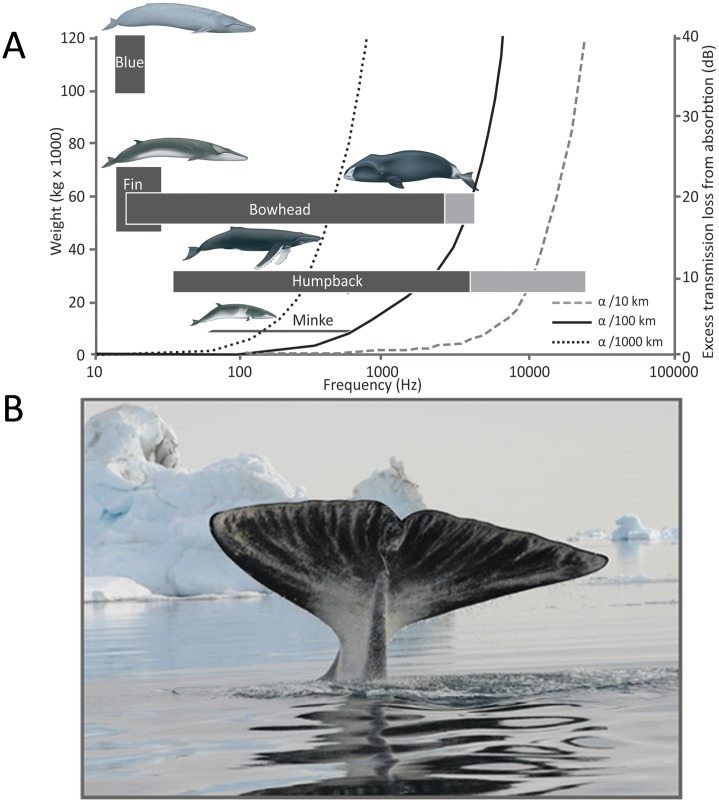
Fundamental frequency of songs and range of body weights (reference IWC) for singing baleen whale species together with the excess transmission loss from absorption (α) at 10 km, 100 km and 1000 km [**45**] **.** A) The grey colour for bowhead whale and humpback whale mark the frequency range of harmonics. References for frequency of song: blue whale [11]; fin whale [13]; bowhead whale [24], [25], [27]; humpback whale [21], [22], [60]; and minke whale [68]. Illustrations by Uko Gorter. B) Bowhead whale *Balaena mysticetus* showing its tail fluke before a dive in Disko Bay, West Greenland (Photo: C. Ilmoni, Qeqertarsuaq Bowhead Research Group).

But what defines the frequency of animal vocalizations? Fletcher (2004) [16] and Gillooly and Ophir (2010) [17] have presented convincing evidence for an inverse relationship between animal size and the peak frequency for sound production. Larger animals in general produce lower frequency signals at higher sound pressures than do smaller animals [16]–[17]. Hence, large animals will generally have a larger active space than small animals for the same power output. Large balaenopterid whales such as fin and blue whales fit such scaling predictions by being the largest marine mammals, and together with the African elephant (*Loxodonta Africana)*, they produce the lowest frequency signals of any studied mammal [11], [13], [18] (Fig. 1A). However, not all mammalian species follow these scaling predictions [19]. For example humpback whales (*Megaptera novaeangliae*), with a body mass of 15–30 tons [20], produce high frequency song notes with fundamental frequencies ranging from 30 to 4000 Hz [21], [22]. Bowhead whales (*Balaena mysticetus*) (Fig. 1B) rival fin whales in size with a body mass of 50–80 tons [20], [23], yet they produce high frequency song notes with fundamental frequencies ranging from 20 to 4000 Hz [24], [25], with centroid frequencies some 4–6 octaves higher than those of the similar sized fin whales (Fig. 1A). Bowhead whales sing during winter and spring [26], [27] and have multiple songs in their repertoire in a given year [28], [29]. Song repertoire includes both simple and complex songs [28], [29], and in some songs, the complexity is achieved by dual sound production by one animal [30].

Thus the vocalizations of bowhead whales, like humpback whales, are produced at much higher frequencies over much broader bandwidths compared to fin whales of similar body mass. Here we explore the consequences of such high frequency vocalizations for the active space of bowhead whales and discuss implications for the evolution of acoustic and mating behaviour in baleen whales.

## Methods

### I. Recordings

Recordings were made in Disko Bay (69o15’ N, 51o25’ W), Western Greenland from March 5 to March 9, 2009. The bay has an average depth of 200 m with a trench in the middle of the bay extending to over 800 m in depth. The average air temperature between February 15 and March 9, 2009, was −17.1±4.0°C resulting in extensive ice cover during the time of the study. Disko Bay has been known to be an aggregation area for bowhead whales for centuries [31]. Every year bowhead whales can be observed close to the shores of Disko Island from mid-February to late May. The area is visited by ∼1200 individuals annually in April and May [32], of which 78% are females [33].

**Figure 2:**
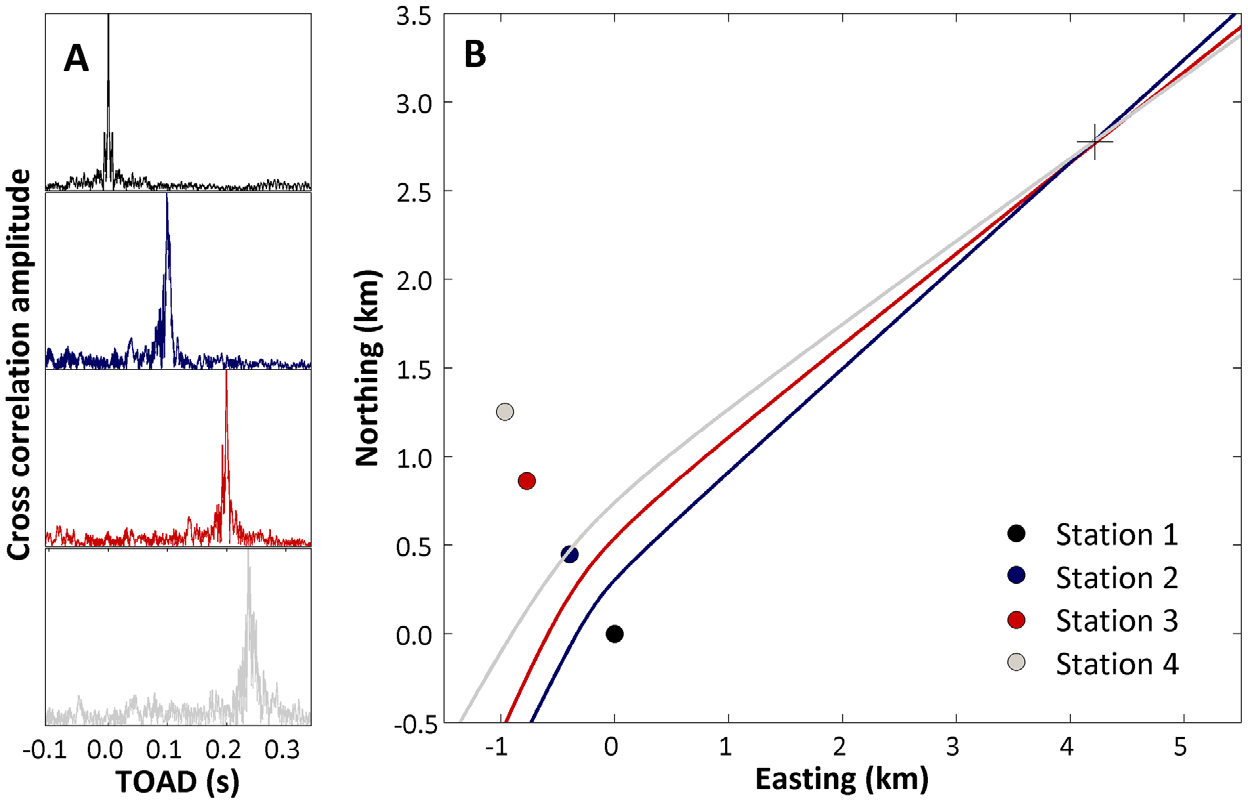
Acoustic localization using a four-channel hydrophone array at four stations separated by about 500 m. A) Cross correlation functions for three stations relative to station 1 (upper panel, an autocorrelation). The peak of each station (stations 2 to 4) indicates the time-of-arrival difference relative to station 1. B) 2D localization plot in a coordinate system (km) referenced to station 1. Each hyperbola indicates all source positions that would result in the time-of-arrival difference measured between station 1 and each of the three other stations. The cross indicates the most likely position of the source as calculated with the method of least squares.

A hydrophone array consisting of four independent receivers was used to record bowhead whale song and ambient noise levels. The receivers were synchronized by using a GPS system that generated timing pulses with 50 µs resolution [34]. At each of four recording stations, a hydrophone was deployed to a depth of 25 m through a hole drilled in the sea ice above a water depth of at least 200 meters. Recording stations were spaced about 500 m apart in a quasi-linear array (see Fig. 2). Each recording station consisted of a B&K 8101 hydrophone (Brüel & Kjær, Nærum, Denmark, sensitivity: −184 dB/V re 1 µPa) connected *via* a custom-built low noise amplifier (40 dB gain, 1 pole high pass at 10 Hz and 4 pole low pass at 25 kHz) to one of the channels of an M-Audio Microtrack II 24/96 digital recorder sampling at 96 kHz (16 bit). The self-noise of this system was measured in a silent room at the Technical University of Denmark to be below Wentz 0 in the frequency range from 0.01 to 10 kHz. All recording chains were calibrated before and after the recordings using a Brüel & Kjær 4228 pistonphone. The GPS timing signal from a frequency-shift-keying (FSK) device [34] was recorded simultaneously on the second audio channel of the M-audio allowing for post-recording derivation of geo-referenced position and absolute timing throughout the recordings. Due to the very low temperatures, all equipment was run on lithium-ion battery cells.

The data collection for this study included the collection of passive acoustic data from bowhead whales and background noise together with a playback experiment of a test signal for sound attenuation. In Greenland there currently exists no legislation for the collection of passive acoustic data or sound playback in connection with a scientific project and therefore no permits are required. The project was conducted at the Arctic Station, University of Copenhagen.

### II. Song Classification

Bowhead whales have a large and dynamic vocal repertoire making the classification of their vocalizations challenging. A song in bioacoustics is defined as a series of stereotyped notes that are repeated in a predictable pattern [35], [36]. The complexity of song varies greatly between species [9] and in some species also between seasons and individuals [37]. Calls in contrast are generally shorter in duration, lower in frequency and simpler in structure than song notes [3], [9], [33], and in birds they are produced by both sexes throughout the year serving a particular function such as alarm calls and contact calls [37].

Bowhead whales produce a variety of different simple frequency modulated (FM) and complex amplitude modulated (AM) calls [24, 25, 27, 38, and 39] as well as both simple and complex songs [24]–[26], [28], [29]. Calls can sometimes be produced as sequences that some authors refer to as simple song [24] and others as song-like calling [39]. In the literature, bowhead tonal signals with frequencies below 500 Hz are most often referred to as calls[24], [25], [27], [38]–[41] and sometimes as song notes (when produced as a sequence) [24], [27], [29], [40], whereas all tonal signals with energy above 1 kHz produced in a sequence are categorized as song notes[24]–[29], [40].

The stereotyped, tonal vocalizations with broad frequency ranges, which were produced in the sequences we recorded, fulfil the definition of simple song. In addition, multiple individuals produced the same sequence and the sequence remained unchanged as part of the repertoire at least until April 1, 2009 (unpublished data) further supporting the classification of these signals as simple song. We therefore denote the recorded vocalizations as song throughout the text.

### III. Data Analysis

Song notes that were chosen for estimation of source level had to satisfy the following criteria: no interference from other sounds, an in-band signal-to-noise ratio (SNR) >10 dB and be recorded simultaneously on all four recording stations. The 2D location of the sound source was estimated by the time-of-arrival differences of the same signal on the four receivers [42], [43]. The time-of-arrival difference was determined by cross-correlating the signals on three receivers with that on a reference hydrophone (recording station 1, Fig. 2). The source location was determined along hyperbolic lines derived from the time-of-arrival differences between the receivers and their spatial geometry [42]. With four receivers, this resulted in three independent hyperbolas [43]. The location of the sound source relative to the hydrophone array was estimated by solving the three hyperbolic equations with the method of least-squares [42], [44]. An example of localization is shown in Fig. 2. The apparent source level (ASL) is the sound level at 1 m from the source (the whale) at an unknown angle from the acoustic axis [34]. We calculated the ASL from the received level (RL) by adding the calculated transmission loss (TL) estimated from geometrical spreading and frequency dependent absorption using the equations of Kinsler *et al*. (2000) [45]. To compute the speed of sound, we recorded salinity and temperature in the water column from 1 to 180 m at 1 m intervals using a Seabird SBE-25-01-CTD (Sea-bird Electronics, Inc., WA, USA). The measured temperature was −1.7°C and the salinity 3.3% at the depth of the hydrophones (25 m) resulting in an estimated sound speed of 1439 m/s.

Short range spreading loss was measured by projecting a 10 ms sweep with a frequency range from 400 Hz to 6 kHz from a Lubell LL916C underwater loudspeaker (Lubell Labs Inc. Columbus, Ohio USA) at a depth of 10 m in three different sessions. The measuring hydrophones were at 10 m, 50 m and 500 m from the source and the FSK signal was used for timing.

Before analysis, all song data were band pass filtered between 0.1 and 4 kHz (first order Butterworth). The ASL was calculated as peak-to-peak (dB re 1 µPa pp @ 1 m), root-mean-squared (dB re 1 µPa rms @ 1 m) and energy flux density (efd, dB re 1 µPa2s @ 1 m) [7], [44]. In addition to sound level measurements, the duration (Dur, s), maximum frequency (F_max_, Hz), minimum frequency (F_min_, Hz), centroid frequency (F_c_, Hz), peak frequency (F_peak_, Hz) and rms bandwidth (BW_rms_, Hz) were calculated for each signal using an FFT size of 4096. Signal duration was defined as the duration that included 98% of the signal energy in the selection window. Minimum and maximum frequencies of the signal were defined as the lowest and highest −10 dB points in the power spectrum, and peak frequency corresponds to the frequency in the signal with maximum energy. The centroid frequency divides the signal into two parts of equal energy on a linear scale. The BW_rms_ was calculated as the spectral standard deviation around the centroid frequency [44].

**Figure 3:**
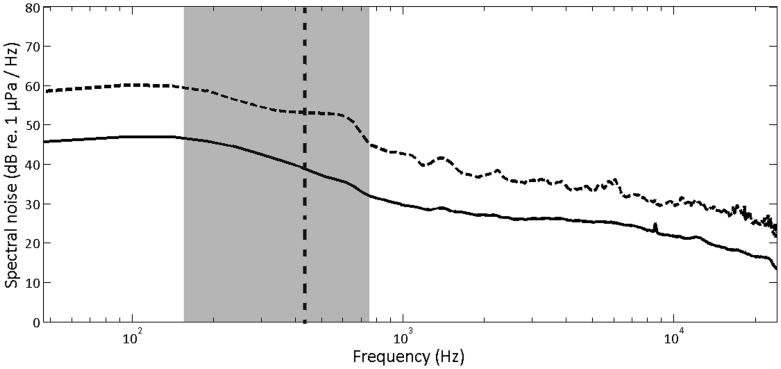
Ambient spectral noise level in Disko Bay at 25 m depth from March 6 and 9 2009. The solid line shows the mean ambient noise level (n = 720) and the dashed line shows the positive standard deviation for these values. The vertical dashed black line marks the centroid frequency of 444 Hz of bowhead whale song notes and the grey area indicates the 285 Hz root-mean-square (rms) bandwidth of these signals. The spectrum level of the masking noise is about 40 dB re 1 µPa^2^/Hz in the bandwidth of a bowhead whale song note.

To estimate the conspecific detection threshold for the song notes, we assumed that signal detection by a whale was limited by the background noise, as is the case for most mammals in the frequency range at which they vocalize [46], [47]. A full picture of the auditory scene and the fluctuating ambient noise over the singing season of bowhead whales would require continuous recording with autonomous units for three months. Due to the very harsh conditions of the ice covered Disko Bay such an approach was not feasible in 2009, and therefore we estimated ambient noise levels from recordings made through ice-holes. Due to the active calling of several whales, we carefully identified 0.5s segments in the recordings with no detectable calls for noise analysis, amounting to a total of 6 minutes from March 6 and 9. A PSD (Power Spectral Density, Welch method) analysis was performed to provide the spectral noise density in dB re 1 µPa^2^/Hz. Each 0.5 s recording was subsequently cut into segments of 1024 samples overlapping by 75%. Data from each 1024 sample element were then combined in an array to form the basis for the noise statistics shown in figure 3. A bandwidth of 284 Hz (the mean BW_rms_ of the call, see the results) over a 0.5 s noise measurement gives a 99% confidence interval of ±1 dB [48].

All analyses were made with custom-written scripts in *MatLab.5* (The Mathworks, Inc. Natick, MA, USA).

## Results

### I. Characteristics of Song Notes

**Figure 4:**
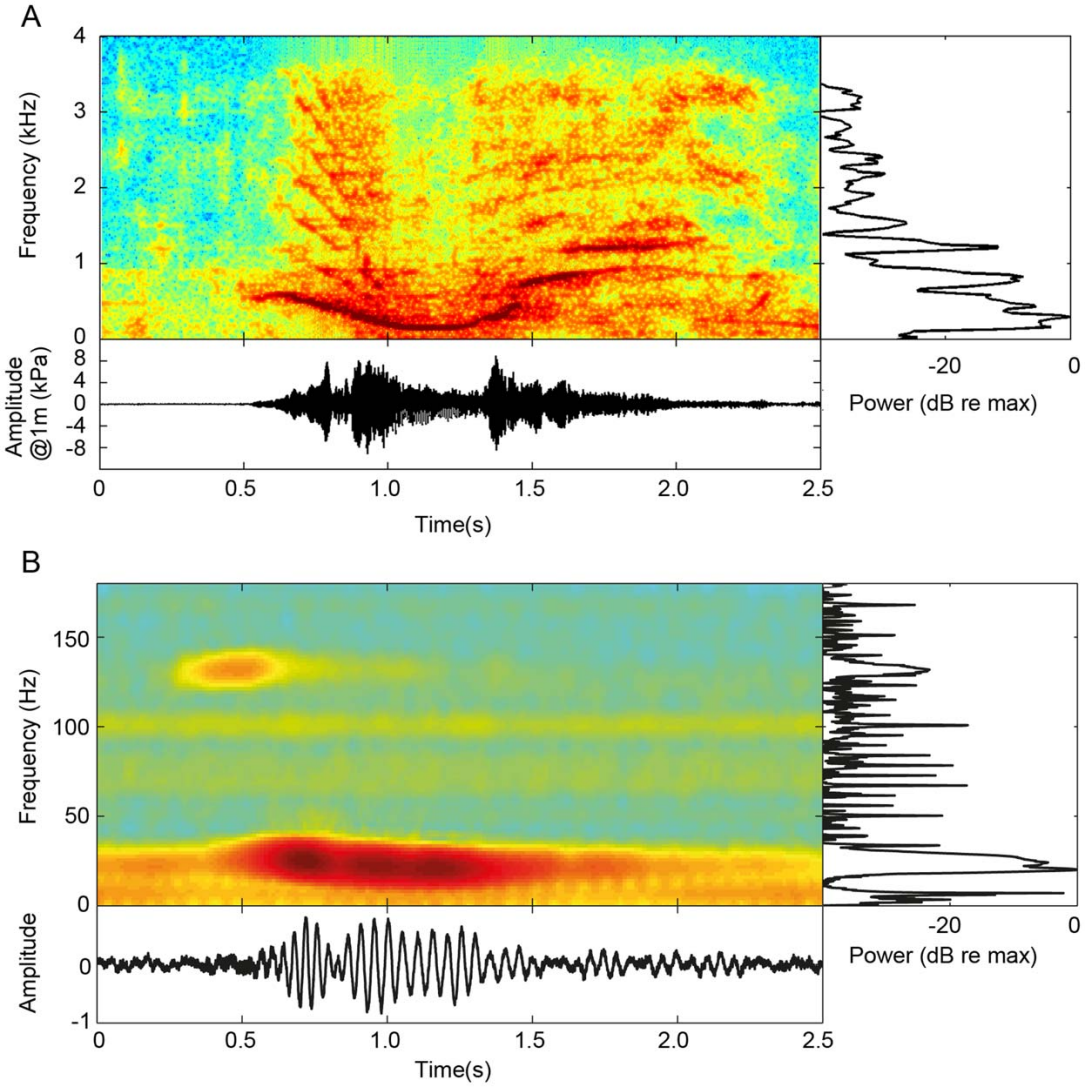
Spectrogram (down-sampled to 8 kHz, window size 256 samples with 95% overlap, fft size 512 with a factor two spectra interpolation), oscillogram (below) and power spectrum (right, Welch power spectral density estimate with a window size of 256 samples) of a bowhead whale song (A) and a fin whale song note (B) (data from Simon et al. 2010 [**53**] **).** The distance to the bowhead whale making the song note is shown in Figure 2. The song consisted of repetitions of this single note. The frequency of the fundamental ranged from 104 Hz to 1356 Hz (Table 1).

The bowhead whale was the only baleen whale species present in Disko Bay at the time of our recordings. Bearded seals (*Erignathus barbatus*) were the only other marine mammals vocalizing during the total of 5 h 5 min of recordings. Out of this total, 2 h 45 min contained bowhead whale vocalizations composed of one stereotyped note that was repeated 7–25 times in a simple song (Fig. 4A). A total of 142 song notes as exemplified in figure 4A had a SNR that allowed for analysis and of these 35 song notes, presumably produced by one individual, fulfilled our criteria for estimating source level. These were recorded on 6 March 2009 on all four recording stations (Fig. 2). The mean ASL was 185±2 dB re. 1 µPa rms @ 1 m. The fundamental frequency of these notes ranged from 104±14 Hz (F_min_) to 1356±102 Hz (F_max_), and was generally comparable to the song notes that could not be localized in terms of duration, centroid frequency and spectral parameters (Table 1).

**Table 1:** Acoustic parameters of song notes.

Song notes		Dur (s)	Fmin (Hz)	Fmax (Hz)	Fc (Hz)	Fpeak (Hz)	BWrms (Hz)	R (m)	TL (dB)	RLpp (dB re µPa)	RLrms (dB re µPa)	RLefd (dB re µPa^2^s	ASLpp (dB re µPa	ASLrms(dB re µPa	ASLefd (dB re µPa^2^s
Localized (n = 35)	Mean	1.5	104	1356	444	468	284	5333	75	130	110	112	205	185	186
		(0.2)	(14)	(102)	(48)	(197)	(71)	(295)	(1)	(3)	(3)	(2)	(3)	(2)	(2)
Others (n = 107)	Mean	1.5	122	1235	465	403	361	NA	NA	125	106	108	NA	NA	NA
		(0.2)	(28)	(248)	(90)	(273)	(114)	NA	NA	(5)	(5)	(5)	NA	NA	NA
T-test (df 141)	p (two-tail)	0.37	1.41^(−6)^	0.00	0.19	0.00				2.64^(−5)^	1.92^(−5)^	2.89^(−5)^			
	Level of significance	>0.05	<0.05	<0.05	>0.05	<0.05				<0.05	<0.05	<0.05			

Localized = song notes fulfilling the criteria for source level estimation. Others = song notes with equally high quality, but were unable to be localized. (Dur, s) = duration, F_max_ (Hz) = maximum frequency, F_min_(Hz) = minimum frequency, F_c_ (Hz) = centroid frequency, F_peak_ (Hz) = peak frequency, BW_rms_ (Hz) = rms bandwidth, R(m) = distance, TL(dB) = transmission loss, RL = received level (rms = root-mean-squared, pp = peak to peak, efd = energy flux density), ASL = apparent source level referenced to 1 m from the source (whale). Standard deviation is given in parentheses.

**Figure 5:**
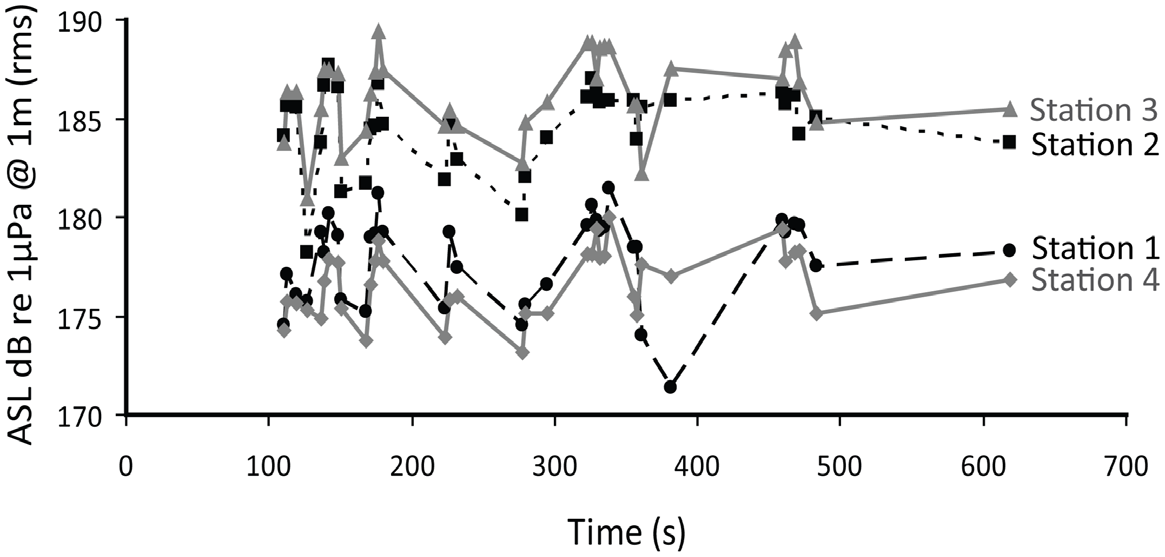
Apparent source level (ASL), defined as dB re 1 µPa (rms) @ 1 m from the whale, for 35 song notes from each of the four recording stations during a song session presumably produced by one individual at 5333±295 m from the centre of the array.

Figure 5 shows the back-calculated apparent source level (ASL) of the localized song notes as a function of time. As shown in Fig. 5, the source level is fluctuating over time. However, these fluctuations are almost synchronized among the stations. The received levels are consistently higher at stations 2 and 3 at the centre of the array compared with stations 1 and 4 situated in the far ends of the array (Fig. 2). Blackwell et al. (2012) [49] found that bowhead whale calls were slightly directional in that the calls were on average 3.3 dB and 3.9 dB (two different data collection set ups) stronger in front of the whale than behind it. The difference in the received levels of song notes was about 10 dB when comparing the weaker stations (1 and 4) with the stronger stations (2 and 3, Fig. 5). This is about three times as much as the source level difference due to directionality reported by Blackwell et al. (2012) [49]. The 1500 meter aperture of the array corresponds to approximately 18 degrees of the full circle around the calculated position of the sound source/whale (see Table 1). Thus the differences in received levels are most likely the result of obstacles, such as icebergs, blocking the direct path of the sound for stations 1 and 4 and not directionality of the vocal structures in the whale. We therefore chose the received levels recorded at stations 2 and 3 for estimating the apparent source level of the bowhead whale song.

### II. Ambient Noise and Sound Velocity

We analysed a total of 6 min of ambient noise from two different days. To estimate the masking noise level that would determine the detection threshold, we summed the ambient spectral noise over the mean BW_rms_ of 284 Hz around the mean centroid frequency of 444 Hz. The mean spectral noise level in that frequency band was 40 dB re 1 µPa^2^/Hz (Fig. 3) resulting in an estimated detection threshold of 65 dB re 1 µPa (rms) (40+10log_10_ (284)), assuming an SNR of 0 dB for detection (Fig. 3).

The sound velocity profile (SVP) was calculated from CTD data. The sound velocity was constant at about 1439 ms^−1^ to a depth of 55 m below which it started to increase gradually resulting in a maximum velocity of about 1462 ms^−1^ at 180 m depth, the maximum depth of our measurements. Thus, the SVP was weakly upwards refracting [50], which can form a surface duct depending on the depth of the receiver, the depth of the source and the frequency of the propagated sound. In this case, use of a geometric spreading model becomes inaccurate for estimating transmission losses over longer ranges. However, for the localization of the whales at around 5 km range, such ducting is unlikely to render transmission loss that deviates much from spherical spreading and, thus, will provide reliable estimates of source level [50]. This notion was supported by short-range transmission loss measurements over a 500 meters range that rendered the expected spherical spreading loss for a sweep covering the song note frequencies of the whales. However, it may be a different issue for estimation of a large active space; a problem we will return to in the discussion.

## Discussion

### I. Active Space of Measured and Predicted Bowhead Song Notes

Blue and fin whale acoustic signals, which approach levels of around 190 dB re 1 µPa rms for about 1 second, are among the most energetic communication signals of any known animal. These powerful signals in combination with very low absorption at 15 to 20 Hz provide the vocalizations of blue and fin whales with the potential to be detectable across entire ocean basins [10]. However, blue and fin whales produce low frequency songs more than 4 octaves lower than the centroid frequency of the high frequency song notes of humpback and bowhead whales (Fig. 1A) raising the question of what are the active spaces for these high frequency singers? In an attempt to answer that question for bowhead whales, we have measured the source levels and spectral characteristics of bowhead whale spring song to address implications of high frequency singing for the acoustic and social behaviour of this large Arctic balaenid.

We measured a mean song source level of 185 dB re 1 µPa (rms) @ 1 m, which is comparable to previous source level estimates of 158–189 dB re 1 µPa @ 1 m of songs and calls recorded from Bering Sea bowhead whales, provided that they were also rms values [25], [40], [51]. The source levels of song notes from fin and blue whales have been reported to range between 180 to 193 dB re 1 µPa (rms) @ 1 m [11]–[14], and are thus comparable with the source level estimates presented here, ranging from 178 to 188 dB re 1 µPa (rms) @ 1 m. The major difference in the vocalizations of fin whales and similar sized bowhead whales is thus not the level, but the frequencies and bandwidths over which the songs are produced. Fin whales produce a 1 second note in which essentially all the acoustic energy is concentrated in a narrow frequency band around 20 Hz [13]. Bowhead whales, on the other hand, produce 1–2 s long song notes that are high-pitched and heavily frequency modulated (Fig. 4A, Table 1) over a frequency band many octaves broader than that of fin whale song (Fig. 4B). Given their size (Fig. 1A), it would be predicted that bowhead whales should sing at frequencies comparable to those of a fin whale, and we will therefore evaluate the consequences of the high frequency song of bowhead whales by comparing with the active space of fin whale song with the same SL in the same area.

To evaluate the consequences for the active space of these two very different bands of singing frequencies, we first assume that both fin whales and bowhead whales are ambient noise limited when detecting acoustic signals [46], [47]. Secondly, we assume that the detection threshold can be estimated from the spectral noise summed over the BWrms of their songs. Estimates of active space are based on the passive sonar equation, and the reliability of that critically hinges on the quality of the input parameters that, for this study, in some cases are well known and for others less so. Consequently, the estimates should be treated with caution, but are nevertheless instructive for comparing active space of high frequency singing in bowhead whales to the very low frequency song of similar sized balaenopterids under the same conditions.

During our study, the ambient noise levels in Disko Bay were very low (Fig. 3) compared to normal open water Wenz curves [52]. This condition probably results from the extensive ice cover essentially eliminating wave noise and effectively preventing ship traffic and the movements of icebergs in the area. Consequently, the masking noise is likely to be as low as it can get in this habitat. For these conditions, the detection threshold of a bowhead whale song note, with a centroid frequency of 444 Hz and a bandwidth (BWrms) of 284 Hz, is probably at best the 65 dB re 1 µPa (rms) estimated here. Fin whales on the other hand vocalize around 20 Hz where the spectral noise in Disko Bay during the recording period was measured to be 45 dB re 1 µPa2/Hz, or some 5 dB higher than that at the centroid frequency of bowhead whale song notes. However, because the BWrms of a fin whale call is only 4 Hz [53], the estimated detection threshold for fin whales under these low noise conditions is only about 51 dB re 1 µPa (rms). So despite lower spectral noise levels at higher frequencies, bowhead whales will have higher detection thresholds than those of fin whales due to the much broader bandwidth over which the song power is distributed. The differences in frequency and bandwidth will also have other consequences for the active space in these two species.

Frequency dependent absorption (α) for a bowhead whale song note with a centroid frequency of 444 Hz is around 2 dB/100 km, but only 0.006 dB/100 km for a fin whale song note at 20 Hz (Fig. 1A). If we apply a spherical spreading loss model of 20log(R)+αR (where R is range in meters and α the absorption coefficient), the bowhead whale song with a source level of 185 dB re 1 µPa (rms) in question here will reach a detection threshold of 65 dB re 1 µPa (rms) at an estimated range of about 400 km.

Using the same spreading model and the same low ambient noise levels, a fin whale could detect a song note at about 5000 km when using a detection threshold of about 51 dB re 1 µPa (rms) and a source level of 185 dB re 1 µPa (rms). Whether the animals can in fact hear each other over such extreme distances hinges on the validity of the input parameters such as the detection capabilities of the whale’s auditory system and the spreading model used. While sound propagation over the short distances in question for the acoustic localization made here is likely very close to spherical spreading loss or 20log(R), such a model is too simplistic for the ranges over which we wish to evaluate active space [50].

The sound velocity profile measured in our recording habitat shows a weak upwards-refracting sound propagation typical of Arctic environments [54]. This will create a near surface sound duct, reducing the transmission loss compared to a 20log(R)+αR model, except for very low frequencies below about 20 Hz whose modes are not supported in the duct [50]. However, the presence of near complete ice cover will add downward reflection to the upwards refraction to form a low-pass filter that at long ranges will provide a much higher attenuation of high frequencies than what can be predicted from the 20log(R)+αR model [50]. Urick (1983) [54] compiled measurements from several studies in the Arctic for ice covered situations and showed that at shorter ranges sound propagates better than spherical spreading would predict, and the opposite at longer ranges. So, for frequencies of 400 to 800 Hz, which cover the centroid frequencies of the bowhead whale song notes (Table 1 and Fig. 4A), the 20log(R)+αR model breaks even at some 60 km and reaches a transmission loss of 120 dB (185 dB –65 dB) at about 130 km from the source [54], giving a more realistic estimate of active space for bowhead song.

Interestingly, the propagation conditions in an ice-covered Arctic sea will also provide poorer propagation conditions of the 20 Hz fin whale song at long ranges, reaching a transmission loss of 134 dB at a range of some 3500 km [54] as opposed to at about 5000 km using the spherical spreading model. For open water conditions with more wave action and noise from moving ice, the noise levels may easily be some 20 dB higher [54], reducing the active space significantly for both species. Thus, the active space calculations presented here are likely overestimates because of the very quiet conditions during our study, and should be treated with caution in the light of the complex and changing mixture of sound propagation conditions and noise levels. However, irrespective of the absolute noise levels, fin and blue whale song notes will have active spaces that are at least an order of magnitude greater than those of bowhead whales for the same source levels.

By sharp filtering we find that the energy content of frequencies above 1 kHz in bowhead whale song notes are at least 20 dB lower than those frequencies below 1 kHz. Using the empirical transmission loss data in Urick (1983) [54], the active space for the high frequencies would be substantially less than 40 km. In addition, multipath propagation and reflections will, over long ranges, provide a blurring effect that will further reduce the information that can be decoded [55]. High frequency components in the form of formants and harmonics that may provide timbre for individual recognition [56], [57] will thus have a much smaller active space than energy around the centroid frequency of some 440 Hz. This reduction in signal entropy with distance due to a low-pass filter effect and multipath propagation may be similar to the situation for some bird species where the low frequency part of the birds call serves as a homing signal at longer ranges and higher frequency components can be used at shorter ranges to extract information about the singer [56], [57]. From the active space estimates here it seems that a bowhead whale residing in Disko Bay (having a radius of some 50 km) under quiet conditions will be able to detect and home in on all singing conspecifics no matter where they are in the bay area, but shorter distances of less than 40 km are needed to decode the full content of the signal that may convey information on individual identity. With an average duty cycle of some 44% resulting from a bowhead whale producing on average 1050 song notes per hour, other singing whales are likely the greatest source of interference for decoding the song of one particular whale in the bay, as is the case for many lekking or chorusing animals (e.g. [58]).

### II. Signal Evolution

The bandwidth and centroid frequencies of bowhead whale and humpback whale vocalizations are much higher than can be expected for an animal that is comparable in body mass with fin whales (Fig. 1A). It may be speculated that selection for a more complex vocal repertoire in an acoustically mediated mating scheme has provided an evolutionary driving force for song with an increased bandwidth as suggested for some songbirds [37], [59]. This can only be achieved by vocalizing at a higher pitch as seen in both bowhead whales [this study] and humpback whales [60]. However, despite power outputs for bowhead whale song that are comparable to those of fin and blue whales, the cost of evolving a complex and elaborate acoustic repertoire is a greatly reduced active space owing to a much higher absorption of sound energy distributed over a broader masking band. Humpback and bowhead whale populations form aggregations with high inter-annual site fidelity [e.g. 31, 35]. Their high frequency and dynamic acoustic repertoire can reach the intended receivers while at the same time facilitate localisation of the emitter, despite this much reduced active space. The similarity of the display strategies of humpback and bowhead whales, which belong to two different baleen whale families, balaenids and balaenopterids, may thus be an example of convergent evolution, where high frequency and complex song has coevolved with relatively small scale breeding aggregations. Fin and blue whales do not have any known aggregation grounds for breeding [61]. Rather with their powerful, narrowband and low frequency vocalizations [62] these whales can reach their conspecifics over long distances at the cost of little potential for relaying identity or behavioural state information.

### III. Energetics of Singing

Given the high duty cycle and powerful output of bowhead whale song notes, it is also relevant to evaluate the energetic costs of such vocalizations. From the calculated source level, it is possible to estimate how much energy an individual is using to produce a given vocalization. The acoustic energy radiated by a source can be expressed as [modified from 54]:
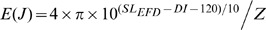
where DI is the directivity index (dB), SL_efd_ is the source energy flux density (dB re 1 µPa^2^s @ 1m), Z is the impedance of the medium (N×s×m^−3^), and 120 is the conversion factor on a dB scale between µPa^2^s and Pa^2^s. Using the mean energy flux density of 186 dB re 1 µPa^2^s @ 1 m for bowhead whale song notes measured here and conservatively assuming omnidirectionality, we calculate that a bowhead whale radiates about 33 J of acoustic energy per vocalization. The sound production efficiency has to our knowledge not been measured in any cetacean species. Jensen et al. (2012) [63] used vocal efficiencies measured in frogs vocalizing in water [64] to conservatively assume a vocal efficiency for bottlenose dolphins of 1%. If we do the same for a bowhead whale producing on average 1050 song notes/h, it will spend some 3500 kJ per hour of active vocalizing (1050 song notes/h × (100×33) J/song note). This number is likely an overestimation as we assume a poor sound production efficiency of 1% and that the song is omnidirectional.

However, even though bowhead whale vocalizations are likely among the most energetic biological sound productions in absolute terms, these spectacular underwater acoustic displays are energetically cheap compared to the field metabolic rate (FMR) of these large animals. Laidre et al. (2007) [65] estimated the FMR of a 60 ton bowhead whale to be 1.2 GJ/day, meaning that the direct costs of sound production constitute maximally 5% of the average FMR during singing. Thus, the powerful and elaborate acoustic display of bowhead whales is likely cheap compared to, for example, visual displays such as breaching [66] or direct physical contact in form of fighting. Nevertheless, acoustic displays can be costly in other ways since time spent vocalizing is not available for feeding, which is also an important part of the bowhead behaviour during spring in Disko Bay [65], [67]. Thus, in late spring bowhead whales must face a trade-off between feeding and acoustic displays to maximize fitness.

### IV. Conclusions

Bowhead whales sing a high frequency song with energy between 100 and 3000 Hz and at a mean centroid frequency of 444 Hz, which is, more than 4 octaves higher than signals of the similar sized fin whales. This high frequency song has likely evolved as a consequence of an acoustically mediated mating scheme selecting for song complexity by driving the song frequency upwards and broadening the bandwidth as has been suggested for many song birds [37], [59]. Despite high source levels of around 185 dB re 1 µPa (rms) @ 1 m, the consequence is that the active space of 130 km of a singing bowhead whale covers an area two orders of magnitude smaller than the area over which the low frequency song of large balaenopterids singing at similar source levels may reach conspecifics. The active space for the higher frequencies in bowhead song is only about 40 km, leading us to propose that bowhead whales may use the low frequency part of the song for homing and the high frequency part to extract information about identity, but only at close ranges. At close range, the broad frequency range of the signal will also enhance the localisation of the emitter by the receiver. We hypothesize that bowhead whales may spatially compensate for their smaller communication range through mating aggregations that co-evolved with broadband song to form complex and dynamic acoustic displays. In spite of high source levels of song notes and a high duty cycle, the energy investment by a singing bowhead whale is less than 5% of the estimated field metabolic rate. Thus the time invested, and not the song itself, is the costly part of these elaborate vocal displays in the Arctic spring where the bowhead whales also feed on copepods to acquire most of their yearly energy intake.
